# QUANTIFYING NURSING HOME CARE QUALITY FOR OLDER HIV AND SEXUAL AND GENDER MINORITY POPULATIONS: A SCOPING REVIEW

**DOI:** 10.1093/geroni/igae098.4059

**Published:** 2024-12-31

**Authors:** Haley Moriarty, Chelsea Wong, Brianne Olivieri-Mui

**Affiliations:** Northeastern University, Northeastern University, Massachusetts, United States; Beth Israel Deaconess Medical Center, Brookline, Massachusetts, United States; Northeastern University, Boston, Massachusetts, United States

## Abstract

Standardized quantitative measures of nursing home (NH) care quality for older people with human immunodeficiency virus (HIV) or sexual and gender minority (SGM) populations are sparce. This scoping review identified the current literature empirically measuring quality of care in NHs for people with HIV (PWH) and SGM populations including, but not limited to, validated measures endorsed by national organizations. The initial librarian-assisted search strategy across four databases resulted in 764 articles. Literature that quantified care quality for HIV (8 studies) or SGM (1 study, 1 report) populations in the NH setting were included. No studies examined the overlap of HIV and SGM populations nor were there studies of general clinical care for either population. The lowest quality was seen with measures of ART prescriptions (n=4/8 studies) and CD4/viral load monitoring (n=2/8 studies). NHs tended to have lower overall care quality (e.g. antipsychotic prescribing, star rating, rehospitalizations) for PWH v people without HIV unless PWH were the majority patient population (n=4/8). One study and one report measured staff/clinicians’ levels of discrimination toward SGM populations finding low willingness to provide care and high rates of refusal to admit SGM patients, respectively. There is insufficient empirical data measuring clinical care quality for SGM using NHs. In conclusion, HIV care quality in NHs has been measured but general clinical care quality for PWH using NHs has not. The growth of the aging HIV and SGM populations demands more research empirically evaluating NH care quality beyond HIV care and including intersecting HIV and SGM identities.

